# A Rare Case of Non-small Cell Lung Cancer With Skeletal Metastasis to the Sartorius Muscle

**DOI:** 10.7759/cureus.56113

**Published:** 2024-03-13

**Authors:** Aniqa Faraz, Sydni Kowalczyk, Mark Hendrixson

**Affiliations:** 1 Internal Medicine, Cumberland Medical Center, Crossville, USA; 2 Oncology, Lincoln Memorial University-DeBusk College of Osteopathic Medicine, Crossville, USA; 3 Oncology, Cumberland Medical Center, Crossville, USA

**Keywords:** non-small cell carcinoma, non-small cell lung carcinoma (nsclc), sartorius muscle, lung cancer, skeletal muscle, muscle metastasis

## Abstract

We report an interesting case of skeletal muscle metastasis from lung cancer. Skeletal muscle metastasis is an unusual clinical occurrence and therefore lacks a standardized treatment approach. A 60-year-old female patient initially presented with abdominal pain and was found to have right lung consolidation, two hepatic lesions, and a lesion to the sartorius muscle. Initially treated as pneumonia, questions arose as to the lesion to the liver as well as the sartorius muscle. The primary site of malignancy was initially questioned due to the large size of the two hepatic lesions, with differential diagnoses including lung or hepatic origin. The sartorius muscle biopsy confirmed the presence of an adenocarcinoma lesion, consistent with non-small cell lung cancer (NSCLC).

## Introduction

Despite significant advancements in the realm of cancer therapy, lung cancer continues to be one of the most lethal malignancies, with adenocarcinoma being its most prevalent subtype. Metastasis of lung cancer to the skeletal muscles is an exceedingly rare occurrence and may present with or without symptoms. The iliopsoas and paraspinal muscles are the most commonly affected sites [1]. It is usually detected incidentally or late in the course of the disease. However, with the introduction of PET-CT, the detected cases of muscle metastasis have increased in number [2]. The purpose of writing this case study is to elaborate on a case with muscle metastasis from lung cancer that was detected incidentally on CT imaging and how it directed the course of disease diagnosis and therapy as the muscle lesion was biopsied, confirming the diagnosis of metastatic lung cancer.

## Case presentation

**Figure 1:**
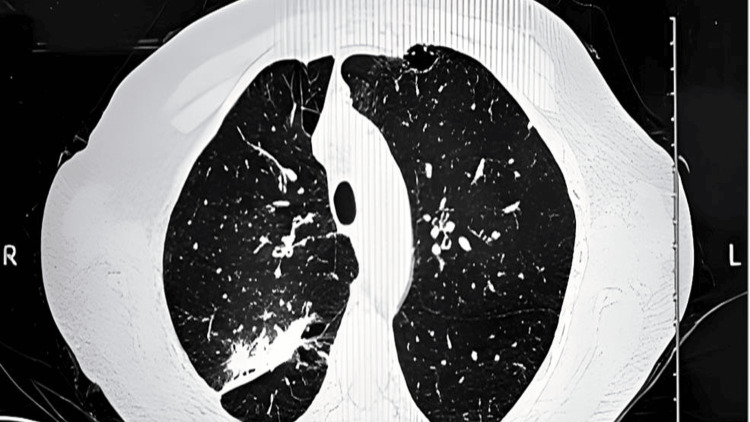
Axial CT chest image demonstrated a mass-like irregular consolidation in the upper lobe of the right lung.

A 60-year-old female smoker with a significant smoking history presented to her primary care physician (PCP) due to generalized weakness. The physical examination revealed right upper lung crackles and hepatomegaly. A low-dose, non-contrast screening lung CT scan, warranted by her smoking history, identified a spiculated mass-like irregular consolidation in the right upper lung lobe (Figure 1).

**Figure 2:**
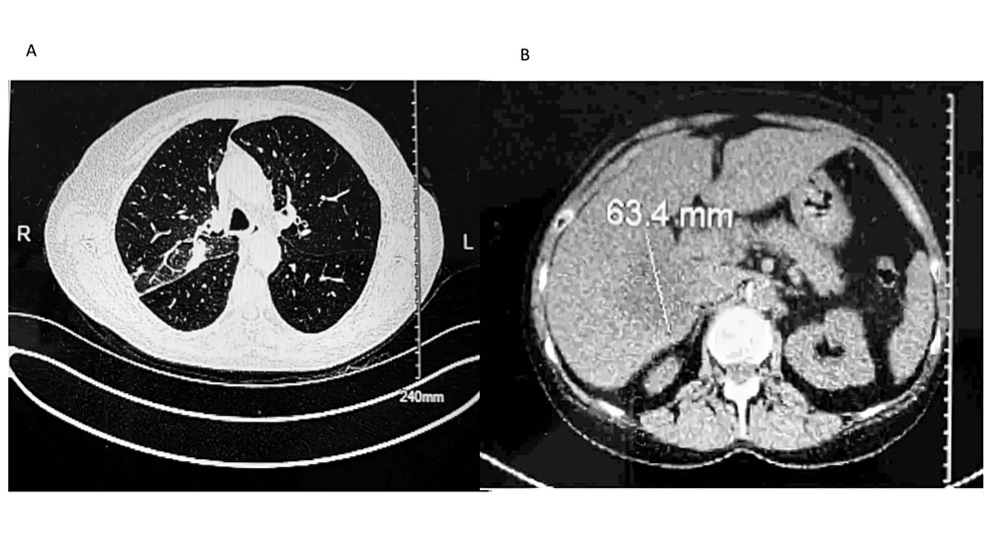
The CT chest without contrast showed right lung consolidation or mass (2A), and hepatic mass (2B).

The patient traveled out of state after the initial consultation, which unfortunately resulted in a temporary discontinuation of her medical care. Upon her return, about three months later, a follow-up CT chest without IV contrast displayed an increased size of lung consolidation and a 63.4mm hepatic metastatic lesion (Figures 2A-2B). The PCP referred the patient to the oncologist. 

**Figure 3:**
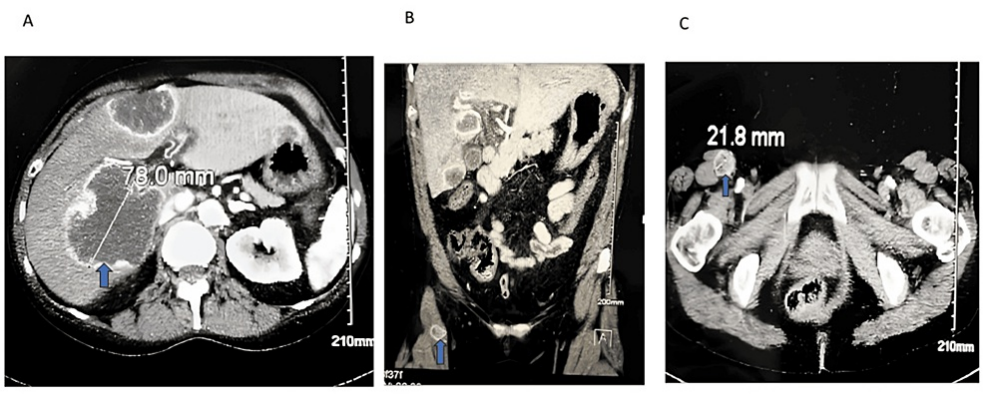
A CT scan of the abdomen and pelvis with IV contrast showed a large hepatic metastatic lesion (3A and 3B) and a right sartorial muscle lesion measuring 21.8mm (3C).

Before the appointment could occur, the patient presented to the emergency department with nausea, vomiting, right upper quadrant pain, generalized weakness, bilateral lower extremity edema, and a persistent lack of energy over the past few months. The physical examination revealed crackles in the lower lobes of both lungs, right upper quadrant abdominal tenderness, and bilateral lower extremity edema. A head CT scan showed no acute intracranial abnormalities. A CT scan of the abdomen and pelvis with IV contrast revealed two hepatic masses, the largest measuring 78mm, and a 21.8mm lesion within the right sartorius muscle (Figures 3A-3C).

The oncologist recommended the biopsy of the sartorius muscle, as he considered it to be the least risky option. This consideration was because of the patient's concurrent treatment with therapeutic Lovenox for an extensive deep vein thrombosis, diagnosed via bilateral lower extremity Doppler ultrasound during this hospitalization.

**Figure 4:**
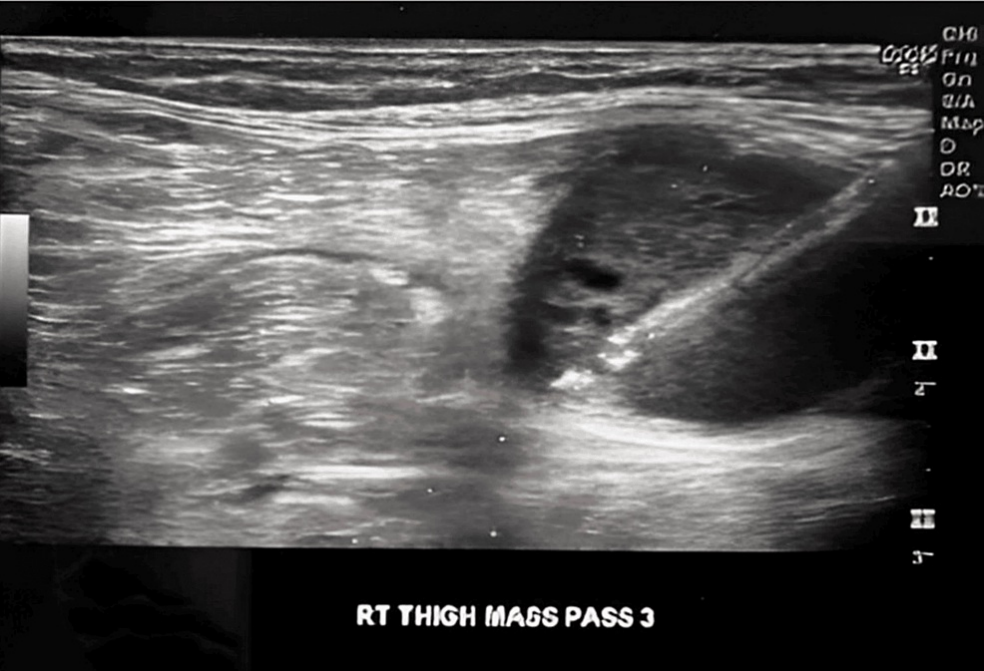
Ultrasound image while obtaining a biopsy of the sartorius muscle

An interventional radiologist got a biopsy of a 2.5cm heterogeneous hypoechoic mass in the right thigh (Figure 4). A follow-up outpatient appointment with the oncologist was scheduled upon discharge from the hospital. 

**Figure 5:**
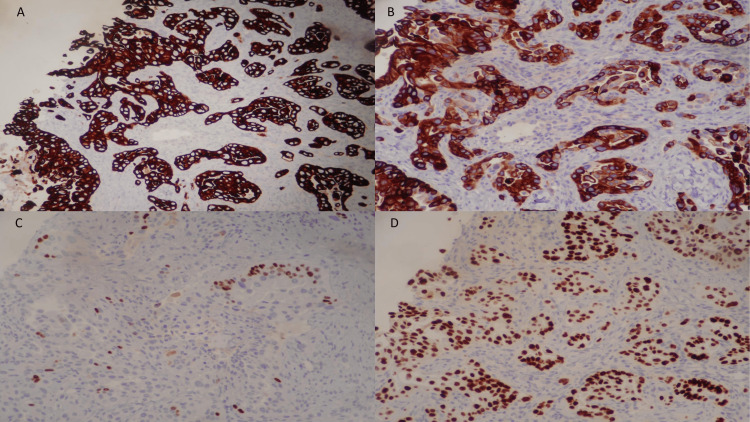
Immunohistochemical slides were positive for A) cytokeratin 7 (cytoplasmic), B) cytokeratin 5/6 (cytoplasmic), C) p63 partial positive (nuclear), and D) TTF-1 (nuclear). TTF-1: thyroid transcription factor 1

At her follow-up appointment, the oncologist reviewed her tumor marker levels and biopsy reports. The alpha-fetoprotein (AFP) level was 3.4ng/ml (nanograms per millimeter) in the normal range, and the carcinoembryonic antigen (CEA) level was elevated to 3.6ng/ml The pathology report confirmed a moderately differentiated adenocarcinoma consistent with pulmonary origin. The cytology report showed rare cohesive clusters of large malignant cells showing some features of squamous differentiation. The immunohistochemically stained slides were positive for cytokeratin 7 (cytoplasmic), cytokeratin 5/6 (cytoplasmic), protein 63 (p63) partial positive (nuclear), and thyroid transcription factor 1 (TTF-1) (nuclear), as shown in Figure 5.

A subsequent PET scan, conducted at an outside facility, revealed hyper-metabolic lesions across various sites, including the right lower lung lobe, right posterior pleura, right hilum, right side of the esophagus, left lower lung lobe, extensive liver metastases, as well as lesions in the right gluteus muscle, right inguinal region, and right scapular region. The patient was ultimately started on chemotherapy for stage IV lung adenocarcinoma. As the muscle lesion was asymptomatic, treatment was not required for the metastatic lesion in this case. The patient passed away within a year of this diagnosis from hypercoagulability-related complications, pulmonary embolism, and stroke.

## Discussion

Muscle metastasis with primary malignancies is a rare occurrence. Lung cancer is one of the primary malignancies that can metastasize to the muscle tissue. Other primary malignancies with muscle metastasis include the pancreas, thyroid, stomach, and kidneys. Muscle metastasis is found in approximately 0.8% of autopsied cancer patients [3]. The relative rarity of these muscle lesions is because of the fluctuating blood flow, the high intramuscular pressure during contractions, and the muscle's high metabolic activity. Vascular emboli from neoplasms are believed to be responsible for muscular metastases and typically manifest as tender nodules or infiltrates, accompanied by swelling [4].

Different primary malignancies metastasize to different locations. For instance, lung cancer commonly metastasizes to the extremities, while breast cancer often spreads to the extraocular muscles [1]. There are three different pathophysiological mechanisms to explain muscle metastasis from malignancies, and it also depends on the type of malignancy and its location. It can be via an arterial, venous, or lymphatic route. The most common venous route is through the paravertebral venous plexus, as this venous plexus has multiple connections to the inferior vena cava and mesenteric venous system. The third way is metastasis through lymphatic spread, especially muscle metastasis to aberrant lymph nodes like in the psoas muscle. We suspect lymphatic spread to the sartorius muscle in our case [5].

There are two main indications for the resection of skeletal muscle metastases. This includes drug-resistant pain caused by the lesion or as a diagnostic measure where non-small cell lung cancer (NSCLC) is suspected but has not yet metastasized, and is deemed a resectable lesion [4].

Muscle metastases indicate a poor prognosis, signifying the aggressive nature of the primary tumor. The treatment plan depends on the number of lesions present. If solitary and symptomatic, a lesion may be excised, followed by chemotherapy. In cases with multiple lesions, local radiotherapy combined with adjuvant chemotherapy may be required [3].

In our case, although the CT scan detected skeletal muscle metastasis, it is important to note that muscle lesions are more commonly identified with PET-CT scans, which significantly influences cancer management strategies [2].

## Conclusions

To conclude our case report, though skeletal muscle metastasis is rare, sometimes it can be detected on a CT scan, and it can be a less risky location for a biopsy to establish a definitive diagnosis of the primary lesion and can help guide further diagnosis and therapy. As the metastatic lesion was asymptomatic in our case, it was not treated, but otherwise, if symptomatic, it can be treated by radiotherapy or excision. Muscle metastasis should be considered a differential diagnosis in any patient presenting with pain or a non-tender mass in the skeletal muscle with risk factors for malignancy. Imaging modalities such as CT, MRI, or PET scans can be helpful in diagnosis and staging, though a core biopsy provides the definitive diagnosis. A PET scan is an excellent modality with the most diagnostic value to detect these rare muscle metastasis lesions; however, in our case, it was detected on a regular CT scan. Skeletal muscle metastasis may also present a poor prognosis, as in our case, the patient passed away within months of the diagnosis of metastatic lung disease to the muscle. 
